# The impact of urgency of umbilical hernia repair on adverse outcomes in patients with cirrhosis: a population-based cohort study from England

**DOI:** 10.1007/s10029-023-02898-6

**Published:** 2023-11-28

**Authors:** A. Adiamah, A. Rashid, C. J. Crooks, J. Hammond, P. Jepsen, J. West, D. J. Humes

**Affiliations:** 1 National Institute for Health Research Nottingham Digestive Diseases Biomedical Research Unit, E Floor West Block, QMC Campus, Nottingham University Hospitals NHS Trust, Nottingham, NG7 2UH UK; 2 Division of Hepatobiliary and Transplant Surgery, Freeman Hospital, Freeman Rd, High Heaton, Newcastle Upon Tyne, NE7 7DN UK; 3 Department of Hepatology and Gastroenterology and Clinical Epidemiology, Aarhus University Hospital, Aarhus, Denmark; 4 Division of Epidemiology and Public Health, School of Medicine, University of Nottingham, Clinical Sciences Building, City Hospital, Nottingham, NG5 1PB UK

**Keywords:** Cirrhosis, Hernia, Umbilical hernia, Elective, Emergency, Postoperative mortality

## Abstract

**Introduction:**

Umbilical hernia is common in patients with cirrhosis; however, there is a paucity of dedicated studies on postoperative outcomes in this group of patients. This population-based cohort study aimed to determine the outcomes after emergency and elective umbilical hernia repair in patients with cirrhosis.

**Methods:**

Two linked electronic healthcare databases from England were used to identify all patients undergoing umbilical hernia repair between January 2000 and December 2017. Patients were grouped into those with and without cirrhosis and stratified by severity into compensated and decompensated cirrhosis. Length of stay, readmission, 90-day case fatality rate and the odds ratio of 90-day postoperative mortality were defined using logistic regression.

**Results:**

In total, 22,163 patients who underwent an umbilical hernia repair were included and 297 (1.34%) had cirrhosis. More patients without cirrhosis had an elective procedure, 86% compared with 51% of those with cirrhosis (*P* < 0.001). In both the elective and emergency settings, patients with cirrhosis had longer hospital length of stay (elective: 0 vs 1 day, emergency: 2 vs 4 days, *P* < 0.0001) and higher readmission rates (elective: 4.87% vs 11.33%, emergency:11.39% vs 29.25%, *P* < 0.0001) than those without cirrhosis. The 90-day case fatality rates were 2% and 0.16% in the elective setting, and 19% and 2.96% in the emergency setting in patients with and without cirrhosis respectively.

**Conclusion:**

Emergency umbilical hernia repair in patients with cirrhosis is associated with poorer outcomes in terms of length of stay, readmissions and mortality at 90 days.

**Supplementary Information:**

The online version contains supplementary material available at 10.1007/s10029-023-02898-6.

## Introduction

Umbilical hernia is one of the commonest types of herniation through the abdominal wall fascia and affects approximately 2% of the general population [1]. In patients with cirrhosis, umbilical hernia formation occurs in approximately 20%, which further rises up to 40% in those with tense ascites [2, 3]. Some of the major risk factors contributing to the higher occurrence in patients with cirrhosis include: increased intra-abdominal pressure and laxity of the abdominal wall fascia from ascites and muscle wasting due to malnutrition [4].

The definitive treatment of umbilical hernia in the general population is surgical repair. However, in patients with cirrhosis the optimal treatment of an umbilical hernia is less clear. The conservative approach was in the past preferable in this cohort of patients due to the higher risk of perioperative morbidity and mortality, with surgical interventions reserved when complications develop [5]. Gray et al. found that patients with cirrhosis were more likely to undergo emergency umbilical hernia repair than those without cirrhosis (26% vs 4.8%), and their outcomes were poorer after emergency surgery [6]. In studies that have compared nonoperative management to open umbilical hernia repair in patients with cirrhosis, elective operation was preferable to nonoperative management. Those in the nonoperative arm were found to be at an increased risk of progression to complications including strangulation, incarceration [6] or spontaneous rupture from necrosis of overlying skin resulting in the eponymous Flood syndrome [7].

The mortality rate following abdominal hernia repair in patients with cirrhosis was reported to be between 3 and 11% in a recent systematic review that reported on extrahepatic gastrointestinal surgery [8]. However, that review was limited by heterogeneous data and was unable to account for confounders or impact of urgency of surgery [8]. In the general population, umbilical hernia repair itself is a low risk procedure, with in-hospital mortality and 30-day mortality rates of between 0.1 and 0.6% [3, 9, 10]. So the higher mortality reported albeit mostly from single centre studies [11, 12] using small patient numbers may have led to an increase in conservative and expectant management despite existing guidelines supporting surgery [13]. There is a lack of population-based data evaluating adverse outcomes and mortality rates following umbilical hernia repair in patients with cirrhosis, especially beyond the in-patient period [3]. This population-based cohort study evaluated the postoperative outcomes including 90-day mortality following umbilical hernia repair in patients with cirrhosis compared with those without cirrhosis, stratified by the urgency of the procedure and severity of cirrhosis.

## Methods

The study was approved by the Independent Scientific Advisory Committee for Medicines and Healthcare products Regulatory Agency (Protocol 19‐193R).

### Patients and data sources

This population-based cohort study used two linked primary and secondary care electronic healthcare databases from England, the Clinical Practice Research Datalink (CPRD) and Hospital Episode Statistics (HES) database. These databases have previously been described in detail [14–16].

### Study participants

Patients aged 18 years and older undergoing umbilical hernia repair were identified using OPCS codes for umbilical hernia repair from CPRD-HES linked dataset between 1st of January 2000 and 31st of December 2017. Patients were followed up until they died, transferred out of a participating general practice, or for 90 days whichever was earliest.

### Exposed cohort

Patients with liver cirrhosis were defined by the presence of diagnosis or procedure codes related to cirrhosis in either HES or CPRD at any time point prior to the date of surgery or by the 90th postoperative day using a previously validated code list [17]. From CPRD data this included the presence of a Read code for cirrhosis, oesophageal varices and/or portal hypertension. The presence of ICD-10 and OPCS-code related to cirrhosis, varices or treatment for varices were used to define cirrhosis in HES data. It has been shown that more than 90% of patients with a diagnosis in secondary care also have supportive evidence of liver cirrhosis entered in either their death certificate or in their primary care records [18].

### Severity of cirrhosis

All patients with cirrhosis were further classified as having either compensated or decompensated disease using the clinical parameters of the Baveno IV classification [18, 19]. The Baveno IV classification as a surrogate for laboratory-derived indices of severity of cirrhosis has been validated [20, 21] and used in other population-based studies investigating patients with cirrhosis [18, 22].

### Covariates

Gender was reported as male or female. Age was categorised into four groups: 18–49 years, 50–59 years, 60–69 years, and 70 years or older. Comorbidity was classified using the Charlson Comorbidity Index [23] into 0, 1 and ≥ 2 and determined from listed comorbidities from CPRD and HES data up to the date of surgery (excluding cirrhosis). The type of admission was defined as elective or emergency, based on the admission record associated with the operation and patients missing data on urgency of admission excluded. The English Index of multiple deprivation (IMD2015) measures relative levels of deprivation in 32,844 small areas or neighbourhoods, called Lower-Layer Super Output Areas in England with patients classified by their postcodes alone. These scores were categorised into quintiles from 1 to 5 (most to least deprived). Complicated and uncomplicated umbilical hernia presentations were derived from the ICD-10 codes associated with the index admission.

### Outcomes

The outcomes of interest were length of stay, 30-day readmission and 90-day case fatality. The time of first diagnosis of umbilical hernia to the date of repair was also compared between groups. Deaths were defined from linked ONS death registration records and included all deaths occurring on the date of surgery and up to 90 days after umbilical hernia repair.

### Statistical analysis

The basic characteristics of the patients with and without cirrhosis were described using frequencies and percentages for categorical variables, with the Chi-squared tests used for significance testing. The Mann–Whitney *U* tests was used for continuous variables, and medians with their associated interquartile ranges (IQR) presented.

The proportion of patients with a diagnosis of umbilical hernia recorded in primary care records prior to their date of surgery was determined from GP records and compared between patients with and without cirrhosis. The earliest date of diagnosis of umbilical hernia in either primary or secondary care records was subsequently used to define the time lag from the date of first diagnosis of the hernia to the date of emergency umbilical hernia repair.

The crude case fatality rate was calculated from the total number of deaths over the total number of patients in each group. Univariate and multivariate logistic regression analyses were undertaken to estimate the unadjusted and adjusted odds ratios (ORs) of postoperative mortality and their respective 95% confidence intervals. Data management and all analyses, stratified by urgency of admission, were performed using Stata® version 16 (StataCorp, College Station, Texas, USA).

## Results

### Demographics

A total of 22,163 patients were identified from the linked data in England undergoing a primary umbilical hernia repair. Of these, 297 (1.34%) had a diagnosis of cirrhosis, out of which 120 (40.40%) had compensated cirrhosis and 177 (59.60) had decompensated cirrhosis. Overall, the majority of patients were male, especially amongst those with cirrhosis (69.70% vs 62.89% in patients without cirrhosis, *P* = 0.014). Those patients with cirrhosis were older, more comorbid and had higher social deprivation. Additionally, whilst the majority of umbilical hernia repairs were performed in the elective setting in patients without cirrhosis (85.94%), in those with cirrhosis 50.51% were performed in the elective setting (Table 1).

**Table 1:** Summary demographics and operative characteristics of all patients with and without cirrhosis undergoing umbilical hernia repair in England from the years 2000 to 2017

	Cirrhosis	No cirrhosis	*P* value
	*n* (%)	*n* (%)	
Gender			
Female	90 (30.30)	8135 (37.20)	0.014
Male	207 (69.70)	13,731 (62.80)	
Age			
18–49	86 (28.96)	10,944 (50.05)	< 0.0001
50–59	84 (28.28)	4596 (21.02)	
60–69	88 (29.63)	3482 (15.92)	
≥ 70	39 (13.13)	2844 (13.01)	
Median (IQR)	58 (49–66) years	50 (38–62) years	< 0.0001
Charlson Comorbidity			
0	49 (16.55)	13,747 (62.87)	< 0.0001
1	60 (20.27)	4472 (20.45)	
2	187 (63.18)	3647 (16.68)	
Deprivation			
1	39 (13.18)	4280 (19.60)	0.001
2	57 (19.26)	4471 (20.47)	
3	62 (20.95)	4787 (21.92)	
4	60 (20.27)	4379 (20.05)	
5	78 (26.35)	3924 (17.97)	
Method of admission			
Elective	150 (50.51)	18,792 (85.94)	< 0.0001
Emergency	147 (49.49)	3074 (14.06)	
Complicated versus uncomplicated			
Uncomplicated hernia	192 (64.65)	19,280 (88.17)	< 0.0001
Complicated hernia^a^	98 (33.00)	2031 (9.29)	
Undefined/missing	7 (2.36)	555 (2.54)	
Severity of cirrhosis			
Compensated	120 (40.40)	–	
Decompensated	177 (59.60)	–	
Operative approach			
Laparoscopic	6 (2.02)	1566 (7.16)	< 0.003
Open	291 (97.98)	20,300 (92.84)	
Length of stay			
LOS all patients	2 (1–5) days	0 (0–1) days	< 0.0001
Readmission			
30-day readmission	60 (20.20)	1266 (5.79)	< 0.0001

^a^Complicated and uncomplicated umbilical hernia were defined from ICD-10 codes

Assessing patients by urgency of umbilical hernia repair, the differences between patients with and without cirrhosis by gender, age and comorbidity persisted. However, deprivation was only significant in patients with cirrhosis undergoing elective, but not emergency surgery. More patients with cirrhosis in the emergency setting had decompensated cirrhosis (74.83%) compared with those presenting for hernia repair electively (44.67%) (Table 2).

**Table 2:** Basic demographics of patients with and without cirrhosis undergoing elective and emergency umbilical hernia repair

	Elective surgery			Emergency surgery		
	Cirrhosis	Non-cirrhosis	*P* value	Cirrhosis	Non-cirrhosis	*P* value
	*n* (%)	*n* (%)		*n* (%)	*n* (%)	
Gender						
Female	46 (30.67)	6637 (35.32)	0.235	44 (29.93)	1498 (48.73)	< 0.0001
Male	104 (69.33)	12,155 (64.68)		103 (70.07)	1576 (51.27)	
Age						
18–49	41 (27.33)	9667 (51.44)	< 0.0001	45 (30.61)	1277 (41.54)	< 0.0001
50–59	36 (24.00)	3977 (21.16)		48 (32.65)	619 (20.14)	
60–69	55 (36.67)	3015 (16.04)		33 (22.45)	467 (15.19)	
≥ 70	18 (12.00)	2133 (11.35)		21 (14.29)	711 (23.13)	
Median (IQR)	59 (49–66) years	49 (38–61) years	< 0.0001	56 (48–65) years	54 (42–69) years	0.0952
Charlson Comorbidity						
0	25 (16.67)	12,130 (64.55)	< 0.0001	24 (16.44)	1617 (52.60)	< 0.0001
1	33 (22.00)	3796 (20.20)		27 (18.49)	676 (21.99)	
2	92 (61.33)	2866 (15.25)		95 (65.07)	781 (25.41)	
Deprivation						
1	21 (14.00)	3740 (19.93)	0.037	18 (12.33)	540 (17.57)	0.098
2	24 (16.00)	3874 (20.64)		33 (22.60)	597 (19.43)	
3	38 (25.33)	4113 (21.91)		24 (16.44)	674 (21.93)	
4	29 (19.33)	3755 (20.01)		31 (21.23)	624 (20.31)	
5	38 (25.33)	3286 (17.51)		40 (27.40)	638 (20.76)	
Complicated versus uncomplicated						
Uncomplicated hernia	138 (92.00)	18,005 (95.81)	< 0.0001	54 (36.73)	1275 (41.48)	0.509
Complicated hernia^a^	9 (6.00)	318 (1.69)		89 (60.54)	1713 (55.73)	
Undefined/missing	^a^	469 (2.50)		4 (2.72)	86 (2.80)	
Severity of cirrhosis						
Compensated	83 (55.33)	–		37 (25.17)	–	
Decompensated	67 (44.67)	–		110 (74.83)	–	
Operative approach						
Laparoscopic	5 (3.33)	1379 (7.34)	0.060	^a^	187 (6.08)	0.060
Open	145 (96.67)	17,413 (92.66)		^a^	2887 (93.92)	
Length of stay						
Median (IQR)	1 (0–2) days	0 (0–1) days	< 0.0001	4 (2–9) days	2 (1–5) days	< 0.0001
Readmission						
30-day readmission	17 (11.33)	916 (4.87)	< 0.0001	43 (29.25)	350 (11.39)	< 0.0001

^a^Cell numbers < 4, so cannot be reported

### Presentation and duration to surgery

Of all patients undergoing umbilical hernia repair, 67.35% of those without cirrhosis and 54.54% of those with cirrhosis had a diagnosis of umbilical hernia recorded in the GP record prior to surgery.

The proportion of patients presenting for emergency umbilical hernia repair greater than 30 days after initial diagnosis was 42.18% (62/147) in patients with cirrhosis compared with 23.39% (719), *P* < 0.0001, in patients without cirrhosis. This included 47 (31.97) patients with cirrhosis who were seen more than 90 days prior to emergency umbilical hernia repair (Table 3).

**Table 3:** The median duration from time of first diagnosis to date of emergency umbilical hernia repair

Duration (*t*)	Emergency		*P* value
	Cirrhosis	No cirrhosis	
0	35 (23.81)	1100 (35.78)	< 0.0001
1–30 days	50 (34.01)	1255 (40.83)	
31–90 days	15 (10.20)	89 (2.90)	
> 91 days	47 (31.97)	630 (20.49)	

*t* = 0, in patients for whom first date of formal record of diagnosis = date of surgery

Median duration to surgery in patients with cirrhosis, emergency = 6(1–178) days

Median duration to surgery in patients with no cirrhosis, emergency = 1(0–18) days

*P* = 0.0054

42.18% (62/147) were diagnosed more than 30 days prior to their emergency admission date

Patients in the emergency setting had higher proportions of complicated umbilical hernia, 60.54% in those with cirrhosis and 55.73% in those without cirrhosis. In the elective setting, only 6% of patients with cirrhosis and 1.69% of patients without cirrhosis had complicated umbilical hernia.

### Length of hospital stay

Patients with cirrhosis had longer lengths of stay in both the elective and emergency settings than those without cirrhosis (Table 2). However, amongst those with cirrhosis, those undergoing elective repair had shorter median length of stay of 1 (0–2) day than those undergoing emergency repair, 2 (1–5) days. Length of stay was further influenced by the severity of cirrhosis. In patients with compensated cirrhosis, length of stay in the emergency setting was 3 (2–5) days, and 5 (2–10) days in those with decompensated cirrhosis (*P* < 0.0001).

### Readmissions at 30 days

The 30-day readmission rates were higher in patients with cirrhosis than in those without cirrhosis in both the elective and emergency settings, with the highest rate of 30-day readmission (29.25%) found in those with cirrhosis undergoing emergency umbilical hernia repair (Table 2).

The 30-day readmission rates also differed by the severity of the underlying cirrhosis. In those with compensated cirrhosis, 30-day readmission rates after elective surgery was 7.23% compared with 16.42% in those with decompensated cirrhosis. Similarly in the emergency setting, 30-day readmission rates were 13.51% compared with 34.55% in those with decompensated disease. After adjusting for age, gender and comorbidity, patients with decompensated cirrhosis, but not those with compensated cirrhosis, had a twofold (OR 2.43, 95% CI 1.25–4.69) and threefold (OR 3.59, 95% CI 2.33–5.52) increased odds of readmission after elective and emergency umbilical hernia repair, respectively (Supplementary tables 1 and 2).

### Mortality

The case fatality at 90 days following elective surgery in patients without cirrhosis was 0.16% (31/18792) and 2% (3/150) in patients with cirrhosis. Following emergency umbilical hernia repair, the 90-day case fatality was higher in both groups. It was 2.96% (91/18792) in patients without cirrhosis and 19% (28/147) in those with cirrhosis undergoing emergency surgery. Given the low event rate in the elective setting, regression analysis was performed for patients with and without cirrhosis, but not explored for severity of cirrhosis. It showed that in the elective setting, patients with cirrhosis had a 12-fold increased odds of mortality (OR 12.14, 95% CI 4.36–33.84) after adjusting for age, gender and comorbidity (Table 4).

**Table 4:** Mortality following elective umbilical hernia repair at 90 days

	Unadjusted OR		Adjusted OR^a^	
	OR	95% CI	OR	95% CI
Cohort				
Non-cirrhotic	1.0	(ref)	1.0	(ref)
Cirrhosis	12.35	3.73–40.85	12.14	4.36–33.84
Gender				
Female	1.0	(ref)	1.0	(ref)
Male	0.88	0.44–1.76	0.96	0.46–1.98
Age (years)				
18–49	1.0	(ref)	1.0	(ref)
50–59	4.04	0.96–16.89	1.79	0.44–7.34
60–69	7.390	1.91–28.61	3.44	0.98–12.10
≥ 70	28.83	8.52–97.51	9.68	2.99–31.33
No. of comorbidities				
0	1.0	(ref)	1.0	(ref)
1	1.41	0.43–4.59	1.10	0.33–3.70
≥ 2	9.65	4.41–21.09	3.89	1.58–9.62
Deprivation				
1	1.0	(ref)		
2	0.83	0.28–2.46		
3	1.30	0.49–3.41		
4	0.99	0.35–2.84		
5	0.48	0.13–1.87		
Complex				
Uncomplicated	1.0	(ref)		
Complicated	1.0	–		
Missing^a^	10.10	4.38–23.31		

^a^Final adjusted model: adjusted for gender, age and comorbidity

In the emergency setting, where impact of severity of cirrhosis could be explored, patients with compensated cirrhosis had a sevenfold increased odds of mortality (OR 7.35, 95% CI 2.97–18.17) and those with decompensated cirrhosis had a ninefold increased odds of mortality at 90 days (OR 9.51, 95% CI 4.97–18.19) compared with patients without cirrhosis after adjusting for the same confounders (Table 5).

**Table 5:** Mortality following emergency umbilical hernia repair at 90 days

	Unadjusted OR		Adjusted OR^a^	
	OR	95% CI	OR	95% CI
Cohort				
Non-cirrhotic	1.0	(ref)	1.0	(ref)
Compensated cirrhosis	9.04	4.02–20.33	7.35	2.97–18.17
Decompensated cirrhosis	7.28	4.30–12.34	9.51	4.97–18.19
Gender				
Female	1.0	(ref)	1.0	(ref)
Male	0.87	0.60–1.25	1.02	0.68–1.53
Age (years)				
18–49	1.0	(ref)	1.0	(ref)
50–59	3.15	1.22–8.16	2.42	0.87–6.72
60–69	9.06	3.86–21.25	6.45	2.53–16.46
≥ 70	22.40	10.28–48.83	19.93	8.24–48.17
No. of comorbidities				
0	1.0	(ref)	1.0	(ref)
1	5.69	2.80–11.58	3.73	1.80–7.71
≥ 2	15.10	8.00–28.50	5.63	2.92–10.86
Deprivation				
1	1.0	(ref)		
2	1.47	0.75–2.89		
3	1.93	1.02–3.64		
4	1.29	0.65–2.56		
5	1.67	0.87–3.21		
Complex				
Uncomplicated	1.0	(ref)		
Complicated	1.52	1.02–2.25		
Missing^a^	1.58	0.55–4.53		

^a^Final adjusted model: is adjusted for gender, age and comorbidity

## Discussion

### What this study found

This study has shown that patients with cirrhosis undergoing elective umbilical hernia repair had better postoperative outcomes than those undergoing emergency repair. Overall, patients with cirrhosis were older, more comorbid and had higher social deprivation; the latter is suggested to negatively influence health-seeking behaviour. For the patients with a prior diagnosis in GP data, this study has demonstrated that those with cirrhosis have this recorded a median of 41 (IQR 0–247) days prior to the date of emergency surgery. When diagnoses in both primary and secondary care are combined, 32% of patients are diagnosed more than 3 months prior to their need for emergency surgery. Given the poorer outcomes in the emergency setting (in particular, relating to the 90-day case fatality risk), it potentially suggests that patients with cirrhosis who are suitable should be considered for surgery earlier, to reduce the risk of developing complications. The need to utilise the elective pathway is further highlighted by the significant difference in the proportion of patients without cirrhosis, 86% undergoing planned elective repair compared with just over 50% in those with cirrhosis.

The length of stay was longer in the patients with cirrhosis, but highest in the emergency setting. This is probably due to the fact that patients with cirrhosis in the emergency setting were ten times more likely to present with complicated umbilical hernia than in the elective setting. Postoperative readmissions in patients with cirrhosis was higher than in those without cirrhosis and worse in those with decompensated disease. Case fatality in the elective setting was low in those patients without cirrhosis, 0.16% compared with 2% in those with cirrhosis, which corresponded to a 12-fold adjusted odds of mortality in patients with cirrhosis. However, in the emergency setting, mortality was higher for all patients with and without cirrhosis than in the elective setting. It was 2.96% in patients without cirrhosis and 19% in those with cirrhosis. When the severity of cirrhosis was considered in this setting, those with compensated and decompensated cirrhosis had a sevenfold and ninefold increased odds of mortality compared to those without cirrhosis, after adjusting for confounders.

### What is already known

Patients with cirrhosis and in particular those with decompensated cirrhosis have a high risk of umbilical hernia formation, estimated to occur in 20–40% of patients [24]. It has been suggested that the natural history of umbilical hernia is towards complication [25] and evidence from two large population-based studies showed that approximately 26–37% of patients not planned for any surgical intervention receive an emergency repair later [6, 26]. Therefore the combined guidelines by the European and American hernia societies states that there is “acceptable evidence that elective umbilical hernia repair is safe in most patients with cirrhosis and/or ascites.” In our analysis, the case fatality rate of 2% in the elective setting was far superior to the mortality rate of 19% in the emergency setting in patients with cirrhosis [13]. The factors described as associated with poor outcomes are a high Model for End-stage Liver Disease (MELD) score above 15, presence of ascites and albumin level below 3 g/dl, which are constituent definition of decompensated cirrhosis. In our analysis, patients with decompensated cirrhosis in the emergency setting had higher odds of mortality than those with compensated cirrhosis. They also had higher rates of readmission and longer postoperative length of stay. Therefore our study adds to the evidence supporting elective umbilical hernia repair, particularly, in the patient with compensated cirrhosis.

The majority of studies on umbilical hernia repair, including those that informed the hernia societies guidelines, have been limited by the population size [11] and also the inclusion of other types of hernias such as incisional hernias [9, 10] and groin hernias [8, 27], which differ in their underlying pathological processes. Nonetheless, studies that have examined chronic liver disease patients only, found similar poorer outcomes in the emergency setting compared to the elective setting [24], with readmission rates of 27% versus 14% in the elective setting [24]. In our analysis, where we could qualify readmission by severity of cirrhosis and urgency of operation, we found 30-day readmission after elective repair to be 7.23% and 16.42% in patients with compensated and decompensated cirrhosis, respectively, compared with 4.87% in those without cirrhosis. By contrast following emergency repair, 30-day readmission rates were 13.51% and 34.55% in those with compensated and decompensated cirrhosis compared with 11.39% in those without cirrhosis.

### Strengths and limitations

This study is one of the largest datasets reported to date from a population level evaluating outcomes in patients with cirrhosis compared to those without cirrhosis undergoing umbilical hernia repair stratified by urgency of procedure. It has shown better outcomes in the elective setting than the emergency setting and better outcomes overall in those with compensated disease. There are, however, some limitations relevant to all database studies that are worthy of note, especially the importance of coding accuracy of both the case and exposure definitions. We overcame the issue of coding accuracy by including only patients who had both the relevant OPCS codes for umbilical hernia repair and an event date to support that procedure. Additionally, we used a validated algorithm to define cirrhosis in both HES and CPRD data which has been shown to have over 90% concordance when validated against patient notes [18] and defined our outcome of mortality from the ONS data. This provides confidence and reliability in our case definition of cholecystectomy, exposure of cirrhosis and outcome of mortality.

Our patients were also categorised into those with uncomplicated and complicated diagnosis with the specified ICD-10 codes associated with the admission spell related to the operative procedure, allowing us to define patients as having complicated or uncomplicated umbilical hernia. The proportions and distribution of complicated hernia are similar to the findings of complicated and uncomplicated umbilical hernia published elsewhere. Nonetheless, a small proportion of patients (2.54%) lacked this information, but were too small to have affected the overall findings.

The MELD and Child–Pugh scores are two of the most recognised risk stratification tools for defining cirrhosis severity and prognosticating. Unfortunately, the detailed biochemical data required to compute these scores were not available in our dataset; therefore, the validated Baveno IV classification tool was used to categorise patients into two broad categories of compensated and decompensated cirrhosis. Given the spectrum of disease severity within each of compensated and decompensated cirrhosis, it will be imperative for future prospective trials to address the impact of stage of cirrhosis on postoperative outcomes, using either of the MELD or Child–Pugh scores, as well as accounting for some of the patient-related factors such as frailty and performance status that are often used to aid surgical decision making.

### Clinical importance

Patients with liver cirrhosis have a higher incidence of umbilical hernia than the general population, but often not offered equal surgical treatments compared with other patient groups with chronic diseases due to fear of postoperative complications. Whilst operative management of patients with cirrhosis overall is suggested to be growing, the currently available literature is sparse, mostly single centre and therefore recommendations even from the two largest societies are not necessarily followed. In our analysis, whilst the majority of patients without cirrhosis had elective repair, in those with cirrhosis there was a 50:50 split between elective and emergency repair, further supporting this assertion that in this patient group operative management is deferred till complications occur, when their mortality risk is most pronounced.

This study using population-level data has shown that in the elective setting, the risk of mortality in patients with cirrhosis after umbilical hernia repair is low. They also have shorter hospital length of stay and lower rates of readmission. Importantly, it has found that there is a prolonged period between GP diagnoses to emergency presentation where patients could be assessed, undergo prehabilitation and optimised for elective repair before complications develop.

## Conclusion

There is a difference in access of elective surgery in patients with cirrhosis. However, for these patients elective surgery offers better outcomes in terms of mortality risk, length of stay and readmission. Emergency umbilical hernia repair was more likely to be complicated and result in increased length of stay and readmissions with higher 90-day case fatality rates. Prospective, multicentre trials, with more in-depth classification of cirrhosis severity, comorbidity, frailty and performance status are now warranted to validate these findings.

### Supplementary Information

Below is the link to the electronic supplementary material.

Supplementary file1 (DOCX 17 KB)

## Declarations

### Author statement

All authors had access to the data and contributed to the drafting of the paper.
