# Supplementary material for: The impact of urgency of umbilical hernia repair on adverse outcomes in patients with cirrhosis: a population-based cohort study from England
Source: Hernia. 2023 Nov 28;28(1):109–17. doi: 10.1007/s10029-023-02898-6 (PMC10891219; doi:10.1007/s10029-023-02898-6)
Supplement: Supplementary file 1 — Supplementary file1 (DOCX 17 KB) [file 10029_2023_2898_MOESM1_ESM.docx]

Supplementary Table 1: Risk of 30-day re-admission following elective umbilical hernia repair

|  | **Unadjusted OR** | | **Adjusted OR** [*****](https://bjssjournals.onlinelibrary.wiley.com/doi/full/10.1002/bjs.9923#bjs9923-note-0003_20) | |
| --- | --- | --- | --- | --- |
|  | **OR** | **95% CI** | **OR** | **95% CI** |
| **Cohort** |  |  |  |  |
| Non-cirrhotic | 1.0 | (ref) | 1.0 | (ref) |
| Compensated Cirrhosis | 1.52 | 0.66-3.50 | 1.01 | 0.44-2.34 |
| Decompensated Cirrhosis | 3.83 | 2.00-7.34 | 2.43 | 1.25-4.69 |
| **Gender** |  |  |  |  |
| Female | 1.0 | (ref) | 1.0 | (ref) |
| Male | 0.74 | 0.65-0.85 | 0.75 | 0.66-0.86 |
| **Age (years)** |  |  |  |  |
| 18-49 | 1.0 | (ref) | 1.0 | (ref) |
| 50-59 | 1.10 | 0.92-1.32 | 1.16 | 0.96-1.41 |
| 60-69 | 1.48 | 1.24-1.77 | 1.34 | 1.09-1.64 |
| ≥70 | 1.94 | 1.61-2.34 |  |  |
| **No. of co‐morbidities** |  |  |  |  |
| 0 | 1.0 | (ref) | 1.0 | (ref) |
| 1 | 1.63 | 1.38-1.93 | 1.58 | 1.33-1.87 |
| ≥2 | 2.83 | 2.43-3.31 | 2.52 | 2.12-2.99 |
| **Deprivation** |  |  |  |  |
| 1 | 1.0 | (ref) |  |  |
| 2 | 0.93 | 0.75-1.14 |  |  |
| 3 | 1.02 | 0.83-1.25 |  |  |
| 4 | 1.08 | 0.88-1.33 |  |  |
| 5 | 1.18 | 0.96-1.46 |  |  |
| **Complex** |  |  |  |  |
| Uncomplicated | 1.0 | (ref) |  |  |
| Complicated | 1.63 | 1.08-2.47 |  |  |
| Missing* | 1.48 | 1.03-2.13 |  |  |

Supplementary Table 2: Risk of 30-day re-admission following emergency umbilical hernia repair

|  | **Unadjusted OR** | | **Adjusted OR** [*****](https://bjssjournals.onlinelibrary.wiley.com/doi/full/10.1002/bjs.9923#bjs9923-note-0003_20) | |
| --- | --- | --- | --- | --- |
|  | **OR** | **95% CI** | **OR** | **95% CI** |
| **Cohort** |  |  |  |  |
| Non-cirrhotic | 1.0 | (ref) | 1.0 | (ref) |
| Compensated Cirrhosis | 1.22 | 0.47-3.14 | 1.0 | 0.38-2.61 |
| Decompensated Cirrhosis | 4.11 | 2.73-6.18 | 3.59 | 2.33-5.52 |
| **Gender** |  |  |  |  |
| Female | 1.0 | (ref) | 1.0 | (ref) |
| Male | 0.82 | 0.67-1.02 | 0.84 | 0.67-1.05 |
| **Age (years)** |  |  |  |  |
| 18-49 | 1.0 | (ref) | 1.0 | (ref) |
| 50-59 | 0.91 | 0.67-1.27 | 0.78 | 0.57-1.07 |
| **60-69** | 1.47 | 1.08-1.99 | 1.17 | 0.85-1.61 |
| ≥70 | 1.56 | 1.19-2.03 | 1.20 | 0.89-1.60 |
| **No. of co‐morbidities** |  |  |  |  |
| 0 | 1.0 | (ref) | 1.0 | (ref) |
| 1 | 1.52 | 1.15-2.02 | 1.41 | 1.06-1.88 |
| ≥2 | 2.45 | 1.92-3.12 | 2.01 | 1.53-2.63 |
| **Deprivation** |  |  |  |  |
| 1 | 1.0 | (ref) |  |  |
| 2 | 1.27 | 0.88-1.84 |  |  |
| 3 | 1.17 | 0.81-1.68 |  |  |
| 4 | 1.35 | 0.94-1.93 |  |  |
| 5 | 1.55 | 1.09-2.20 |  |  |
| Complex |  |  |  |  |
| Uncomplicated | 1.0 | (ref) |  |  |
| Complicated | 1.03 | 0.83-1.28 |  |  |
| Missing* | 0.91 | 0.46-1.79 |  |  |
